# Regulating the Polarization of Macrophages: A Promising Approach to Vascular Dermatosis

**DOI:** 10.1155/2020/8148272

**Published:** 2020-07-28

**Authors:** Huiling Peng, Dehai Xian, Jiexiong Liu, Shihong Pan, Ran Tang, Jianqiao Zhong

**Affiliations:** ^1^Department of Dermatology, Affiliated Hospital of Southwest Medical University, Luzhou 646000, China; ^2^Department of Anatomy, Southwest Medical University, Luzhou 646000, China; ^3^Office of International Exchange, Affiliated Hospital of Southwest Medical University, Luzhou 646000, China

## Abstract

Macrophages, a kind of innate immune cells, derive from monocytes in circulation and play a crucial role in the innate and adaptive immunity. Under the stimulation of the signals from local microenvironment, macrophages generally tend to differentiate into two main functional phenotypes depending on their high plasticity and heterogeneity, namely, classically activated macrophage (M1) and alternatively activated macrophage (M2). This phenomenon is often called macrophage polarization. In pathological conditions, chronic persistent inflammation could induce an aberrant response of macrophage and cause a shift in their phenotypes. Moreover, this shift would result in the alteration of macrophage polarization in some vascular dermatoses; e.g., an increase in proinflammatory M1 emerges from Behcet's disease (BD), psoriasis, and systemic lupus erythematosus (SLE), whereas an enhancement in anti-inflammatory M2 appears in infantile hemangioma (IH). Individual polarized phenotypes and their complicated cytokine networks may crucially mediate in the pathological processes of some vascular diseases (vascular dermatosis in particular) by activation of T cell subsets (such as Th1, Th2, Th17, and Treg cells), deterioration of oxidative stress damage, and induction of angiogenesis, but the specific mechanism remains ambiguous. Therefore, in this review, we discuss the possible role of macrophage polarization in the pathological processes of vascular skin diseases. In addition, it is proposed that regulation of macrophage polarization may become a potential strategy for controlling these disorders.

## 1. Introduction

Macrophages are a group of innate immune cells coming from peripheral blood monocytes. Owing to their multifunctional activities, macrophages potently work in homeostasis maintenance, inflammation, angiogenesis, wound healing, etc. [1]. In general, macrophages differentiate into two functional phenotypes at the microenvironment signal stimulus, namely, classically activated macrophage (M1) and alternatively activated macrophage (M2); this is known as macrophage polarization [2, 3]. M1, an inflammatory phenotype, is dominated by Toll-like receptor (TLR) 4 ligand, lipopolysaccharide (LPS), or Th1 cytokines [e.g., IFN-*γ*, TNF-*α*, and granulocyte-macrophage colony-stimulating factor (GM-CSF)], being marked by CD40, CD80, and CD86 and possessing proinflammatory, tumoricidal, and antimicrobial activity [4, 5]; inversely, M2 is an anti-inflammatory phenotype polarized by Th2 cytokines [e.g., IL-4, IL-10, IL-13, and macrophage colony-stimulating factor (M-CSF)], specifically expressing the markers of CD163, CD206, CD209, and CD301 and being linked to wound healing, inflammation elimination, angiogenesis, and tumorigenesis [6, 7].

In fact, macrophage polarization often occurs in an inflammatory process and therefore many studies about inflammatory disorders or inflammation-related vascular diseases always focus on these two polarized macrophages [8–10]. Recently, it is found macrophage polarization heavily functions in some immune-mediated inflammatory vascular dermatoses, like Behcet's disease (BD), psoriasis, and systemic lupus erythematosus (SLE) [11–13]. There is an upregulation of M1 polarized macrophages in above disorders, which not only causes disproportions in Th1/Th2 and Th17/Treg cells but also leads to a worsening state of oxidative stress (OS), while M2 polarized macrophages markedly increase in angiogenic disorders, such as infantile hemangioma (IH) [14, 15]. Thus, macrophage polarization imbalance would be a major contributor to these dermatoses, and thereby, regulation of macrophage polarization may be a potential target for vascular skin disease treatment.

## 2. Macrophage Polarization

Macrophage polarization has profound impacts on various physiological and pathological conditions, such as angiogenesis, wound repair, inflammation, and tumorigenesis. Regardless of physiological or pathological process, a serial of signaling pathways and diverse mediators (e.g., cytokines, chemokines, transcriptional factors) are heavily implicated in macrophage polarization. Signals from the local microenvironment are modulated by various receptors on the macrophages to initiate multiple pathways of macrophage polarization.

### 2.1. Related Signal Pathways in Macrophage Polarization

**Figure 1:**
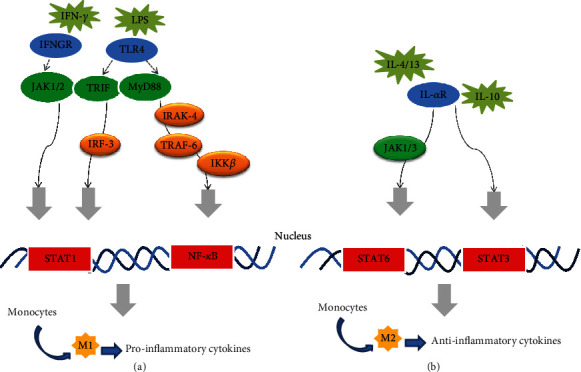
Several signaling pathways mediate in macrophage polarization. (a) M1 macrophage polarization and (b) M2 macrophage polarization are shown with some signal pathways or factors involved in their development. Although this graph displays two categories of macrophage, in fact a dynamic spectrum of polarization often occurs. Abbreviations: IFN-*γ*: interferon gamma; LPS: lipopolysaccharide; IFNGR: interferon gamma receptor; TLR4: Toll-like receptor-4; JAK1/2/3: Janus kinase1/2/3; TRIF: TLR domain-containing adapter protein inducing interferon-*β*; MyD88: myeloid differentiation factor 88; IL-4/10/13: interleukin 4/10/13; IL-*α*R: interleukin receptor; IRF-3: interferon regulatory factor 3; IRAK-4: interleukin-1 receptor-associated kinase 4; TRAF-6: tumor necrosis factor receptor-associated factor 6; IKK-*β*: inhibitor of nuclear factor kappa B kinase; STAT1/3/6: signal transducer and activator of transcription 1/3/6; NF-*κ*B: nuclear factor kappa B.

On exposure to local microenvironment, macrophages convert into two phenotypes (M1 and M2) via activating related signaling pathways. In the process of M1 macrophage polarization, two well-known signals, namely, IFN-*γ* and LPS, are mainly involved [16–18]. After binding to their corresponding receptors (IFNGR and TLR4), IFN-*γ* and LPS recruit the adaptors of Janus kinase 1/2 (JAK1/2), TLR domain-containing adapter protein [interferon-*β* (TRIF) and myeloid differentiation factor 88 (MyD88)], further activating the downstream factors of interferon regulatory factor 3 (IRF3), IL-1 receptor-associated kinase 4 (IRAK-4), TNF receptor-associated factor 6 (TRAF-6), and inhibitor of nuclear factor kappa B kinase (IKK-*β*), ultimately resulting in the activation of signal transducer and activator of transcription 1 (STAT1) and nuclear factor kappa B (NF-*κ*B) [19–21]. These factors contribute to M1 polarization that promotes the expression of inflammatory genes including *TNF-α*, *B lymphocyte stimulator* (*BAFF*), *IL-1B*, *cyclooxygenase 2* (*COX2*), *CXCL9*, *CXCL10*, *IL-6*, and *IL-12p40* [22–24]. On the other hand, IL-4, IL-10, and IL-13, respectively, combine with their corresponding receptors to activate JAK1/3, STAT3, and STAT6 [25–27]. Both activated STAT3 and STAT6 encourage M2 polarization and elicit the production of anti-inflammatory cytokines (shown in Figure 1).

But it is notable that there is a dynamic spectrum of polarization occurring in macrophages and the direction of macrophage polarization is modulated by some special signal pathways. More importantly, the phosphatidylinositol 3-kinase/protein kinase B (PI3K/Akt) pathway and its downstream targets recently emerge as the central regulators of activated phenotype in macrophages [28]. This pathway mediates multiple signals (chemokines, LPS, IL-10, and IL-4) from a variety of receptors mainly involving TLR4 and cytokine receptors (IL-*α*R). The PI3K is initially activated by the above signals via binding to their respective receptors, but the activation of different Akt isoforms (the downstream of PI3K, namely, Akt1 isoform and Akt2 isoform) greatly switches the direction of macrophage polarization. Some studies showed that Akt1 activation could inhibit M1 polarization; conversely, Akt2 activation prevented M2 polarization [29, 30]. Until now, however, the specific mechanisms still remain unclear [28].

### 2.2. Inflammatory Mediators from M1 or M2

Different polarized macrophages perform their unique function via secreting a variety of inflammatory mediators, i.e., cytokines, chemokines, transcriptional factors. These mediators in turn actively participate in macrophage polarization. Basing on macrophage phenotypes, the mediators from macrophages are classified into two categories, namely, M1-derived mediators and M2-derived mediators. M1 activation contributes to the production of proinflammatory cytokines (e.g., TNF-*α*, IFN-*γ*, IL-6, IL-10, IL-23), chemokines (MCP-1, CCL2-4, CXCL8-11, and GM-CSF), nitric oxide (NO), and reactive oxygen species (ROS). Moreover, antigen-presenting molecule MHC-II highly express on M1 and benefit M1 polarization [17, 18]. In the presence of M2 activation, however, the following mediators are markedly upregulated, involving anti-inflammatory cytokines [transforming growth factor-*β* (TGF-*β*), IL-4, and IL-13], chemokines (CCL17, 18, 22, 24, and M-CSF, and proangiogenic factors [vascular endothelial growth factor A (VEGF-A), VEGF-C, platelet-derived growth factor (PDGF),epidermal growth factor (EGF), and basic fibroblast growth factor 2 (FGF-2)] [31–33]. As a result, all these inflammatory mediators further mediate in physiological or pathological process and encourage a normal or morbid switch.

## 3. Macrophage Polarization in Physiological and Pathological Conditions

Macrophage polarization serves as a crucial role in physiology and disease status, such as embryonic development, individual growth, homeostasis maintenance, immunity defence, inflammation, and trauma. At the stage of embryonic development, fetal macrophages display an M2 phenotype and energetically promote angiogenesis, tissue growth, and organ formation, angiogenesis in particular [33]. With the maturation of the innate immune system, the M1/M2 population tends to balance and maintains body homeostasis [34]. In pathological conditions, however, when inflammation occurs, vascular tissue is the major response place where macrophages have a switch from M1 to M2 [35]. M1 actively participates in the initial process of vascular inflammation, which benefits to the establishment of a proinflammatory response. If M1 phase persistently exists, tissue damage would occur. Instead, the sequential presence of M2 macrophages facilitates damage termination and tissue repair in the later phase [35, 36]. However, in many vascular disorders especially in vascular dermatosis, the vascular microenvironment fills with various inflammatory mediators from infiltrated lymphocytes and resident parenchymal cells. The interaction of these cells with the mediators initiates a serial of proinflammatory signals to trigger the persistence M1 polarization. The predominance of M1 vs. M2 would result in extensive vascular damage, abnormal repair, and clinical deterioration. On the contrary, the persistence of M2 polarization leads to the occurrence and development of angiogenic diseases.

## 4. Possible Mechanisms of Macrophage Polarization in Vascular Complaints

**Figure 2:**
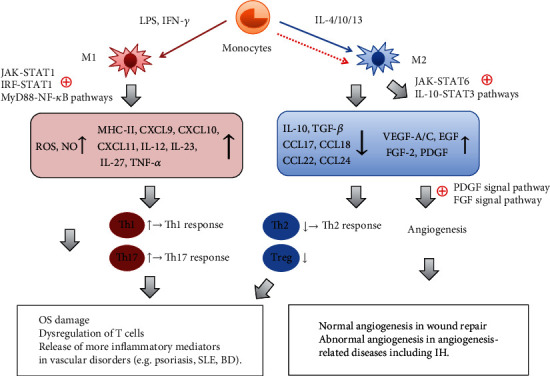
Possible mechanisms of different macrophage polarizations in vascular disorders. Upon the different stimuli, monocytes tend to differentiate into M1 or M2 macrophages via different signaling pathways. In most vascular inflammatory diseases, M1 activation is dominant, whereas M2 activation is relatively inhibited. Through activation of the pathways (JAK/STAT1, IRF/STAT1, and MyD88/NF-*κ*B), activated M1 macrophages release various inflammatory mediators, such as MHC-II, chemokines (CXCL10 and CXCL11), and inflammatory cytokines (TNF-*α*, IL-12, IL-23, and IL-27), to encourage the activation of Th1/Th17 cells and trigger Th1/Th17 response. Apart from that, M1 macrophages produce substantial ROS and NO. As the key factors that regulate the differentiation and chemotaxis of Th2/Treg cells, however, M2-secreted chemokines (CCL17, CCL18, CCL22, and CCL24) and anti-inflammatory cytokines (TGF-*β*, IL-10) markedly decrease along with the inhibition of activated M2. As a result, these events may contribute to the appearance of T cell dysregulation, OS damage, and increased inflammatory mediators in the pathological process of vascular inflammatory disorders or dermatoses, e.g., psoriasis, SLE, and BD. On the other hand, activated M2 macrophages secrete abundant angiogenic factors (e.g., VEGF-A/C, FGF-2, EGF, and PDGF) via stimulating the JAK/STAT6 and IL-10/STAT3 pathways, which not only facilitate normal angiogenesis in wound repair but also promote abnormal angiogenesis in angiogenesis-related diseases including IH by activating the PDGF and FGF signal pathways. Notes: the red and blue solid arrows indicate normal activation, while the red dotted arrow indicates relative inactivation; ⊕indicates “activation,” ↑ indicates “upregulation,” and ↓ indicates “downregulation.” Abbreviations: LPS: lipopolysaccharide; IFN-*γ*: interferon gamma; IL-4/10/13: interleukin 4/10/13; M1: classically activated macrophage; M2: alternatively activated macrophage; ROS: reactive oxygen species; NO: nitric oxide; MHC-II: major histocompatibility complex-II; CXCL9/10/11: chemokine (c-x-c motif) ligand 9/10/11; IL-10/12/23/27: interleukin 10/12/23/27; TNF-*α*: tumor necrosis factor alpha; CCL17/18/22/24: chemokine (c-c motif) ligand 17/18/22/24; VEGF-A/C: vascular endothelial growth factor A/C; EGF: epidermal growth factor; FGF-2: fibroblast growth factor 2; PDGF: platelet-derived growth factor; Th: T helper; Treg: regulatory T cells; OS: oxidative stress; SLE: systemic lupus erythematosus; BD: Behcet's disease; IH: infantile hemangioma

Although the specific mechanism of macrophage polarization in vascular disorders keeps obscure, it is considered that T cell dysregulation, oxidative stress damage, and angiogenesis are probably involved in macrophage polarization-mediated vascular diseases, especially in some vascular skin complaints, e.g., BD, psoriasis, SLE, and IH (summarized in Figure 2).

### 4.1. M1 Polarization Causes T Cell Dysregulation

In vascular inflammation site, macrophages and T cells always coexist and interact with each other [37, 38]. The dynamic equilibrium of M1/M2 benefits to balance inflammatory T cells (Th1 and Th17 cells) and anti-inflammatory T cells (Th2 and Treg cells) in quantity, proportion, and function [39–41]. In certain vascular inflammatory diseases, e.g., psoriasis, BD, and atherosclerosis, however, this balance is broken [42–44]. Under the stimulation from chronic inflammatory signals, macrophages are provoked mainly as M1, whereas M2 activation is relatively inhibited [42, 45]. Activated M1 in turn impels T cell activation and differentiation [46]. CD4+ T cells, as macrophage-stimulated effector cells, usually function as immune modulators by differentiating into various functional subtypes (e.g., Th1/Th2/Th17 cells and Treg cells) [47]. Accordingly, M1-produced chemokines (CXCL9-11) attract Th1/Th17 cells to the vascular inflammatory site [48]; beyond that, some proinflammatory factors from M1 (IL-12, IL-23, IL-27, etc.) encourage Th1/Th17 differentiation and Th1/Th17-derived factor secretion (IL-17A, IFN-*γ*, IL-17F, IL-21, and IL-26) [49, 50]. Consequently, Th1 and Th17 cells promote the progress of vascular inflammation and accelerate vascular tissue damage through secreting above inflammatory cytokines [51–53]. Worse yet, Th1 cells could recruit more M1 to the vascular inflammatory site and prevent M2 activation, thereby forming a vicious circle [54–56]. Just owing to M2 inactivation, the differentiation of anti-inflammatory cells (Th2/Treg cells) is arrested, which may contribute to T cell dysregulation and aggravate vascular tissue damage [57]. Besides, the decrease of Treg cells that skew the macrophages toward M2 would encourage the state of M1 polarization and in turn exacerbate vascular injury [18, 58, 59].

### 4.2. M1 Polarization Aggravates OS Damage

ROS and NO, the crucial mediators of vascular inflammation, are mostly derived from macrophages and potently involved in various vascular inflammatory diseases such as BD, SLE, atherosclerosis, and cancer [60–63]. During the early period of vascular inflammation, ROS and NO from macrophages benefit to eliminate the foreign pathogens [64–66]. As the aggravation of inflammation, however, excessive ROS or NO from macrophages could induce OS and result in OS damage [67]. ROS, the major contributor to OS, are mainly generated by nicotinamide adenine dinucleotide phosphate (NADPH) oxidase in inflammatory responses [68]. Fuchs et al. (2019) indicated that the metabolite profile of M1 displayed an increase in superoxide whereas a decrease in antioxidant; M1 macrophages appeared to consume more NADPH than inactive macrophages or M2 macrophages; instead, increase of taurine (an essential amino acid in biological processes) in M2 macrophages could neutralize and scavenge excessive or harmful ROS [69]. Apart from that, some proinflammatory factors from the vascular microenvironment could drive macrophages to polarize towards M1 [8], further to accelerate the overproduction of ROS/NO and trigger OS [68]. Sustained M1 dominant in vascular diseases/vascular dermatoses (psoriasis, BD, and SLE) in turn induces continuous accumulation of ROS and NO [70–72]. These excessive prooxidants go beyond the antioxidant defense of cells, then to encourage OS initiation and OS state [79–81]; in the persistence of OS state, prooxidants directly hurt tissue biomolecules to form oxidized lipids and denatured proteins, further broke nucleic acids and consequently damage vascular endothelial cells/tissue [73]. Besides, high-level ROS actively work in a feedback-loop switching macrophage to M1 through complex mechanisms involving that ROS-induced TNF-*α* production promotes M1 macrophage activation, and ROS-stimulated MAPK/NF-*κ*B signals mediate proinflammatory genes to reprogram macrophages towards M1, thereby contributing to OS exacerbation and vascular endothelial cells/tissue injury [74–76].

### 4.3. M2 Polarization Promotes Angiogenesis

As one key member of the angiogenic-promoting cells, M2 dominates in wound healing, oncogenic angiogenesis, and blindness-related aberrant angiogenesis [77–79]. M2 macrophages not only facilitate the endothelial cell progenitors to differentiate into endothelial cells [80, 81] but also interfere with all the stages of angiogenesis via release of various proangiogenic growth factors, e.g.,VEGF-A, VEGF-C, PDGF, EGF, and FGF-2 [82–84]. They act as “bridge cells” or “cellular chaperones” that guide the fusion of endothelial tip cells (vascular anastomosis) and facilitate vascular sprouting [85]; they also produce heparinases and plasmin to degrade the extracellular matrix, bind the growth factor to its receptor on endothelial cells, and promote the transmission of growth factor signals [14, 86]. In an experiment on mice, it found that M2-produced IL-10 was a crucial factor that positively derived abnormal angiogenesis, whereas angiogenesis would decrease once IL-10 absence [87]. Moreover, the study *in vitro* showed that angiogenic factor expression on M2 surpassed those expressions on M1 [88]; M2 instead of M1 could induce angiogenesis *in vivo* and *in vitro*, mainly involving the PDGF and FGF signal pathways. Besides, macrophage polarization is closely associated with angiogenesis in tumor growth. Circulating monocytes are recruited into the tumor stroma and then differentiate into tumor-associated macrophages (TAMs). These TAMs release a serial of growth factors (e.g., VEGFA, FGF-2, and PDGF) for endothelial cell proliferation and microvessel formation. Most notably, the phenotype of TAMs is greatly similar to M2; once the phenotype deviates from M2, tumor growth would be suppressed [77]. Therefore, the enhanced proangiogenic activity is generally ascribed to polarized M2. However, M1 scarcely enhances the proangiogenic effect; inversely, it induces endothelial-to-mesenchymal transition and inhibits angiogenesis by releasing inflammatory factors such as TNF-*α* and IL-1*β* [89]. M2-dominated activation, hence, would lead to an amplification of angiogenic effects which plays a major role in some angiogenic diseases such as IH [14].

## 5. Macrophage Polarization in Vascular Dermatosis

Growing evidence supports that unbalanced macrophage polarization occurs in some vascular skin diseases, such as BD, psoriasis, SLE, and IH. As a group of typical vascular inflammatory dermatoses, BD, psoriasis, and SLE collectively exhibit same pathological mechanisms involving M1-mediated immune inflammation, T cell dysregulation, and OS damage. Meanwhile, they own the similar microenvironment which provides macrophage polarization with inflammatory cytokines that induce M1 polarization. In addition, M1-produced factors remarkably ascend in these diseases and positively correlate with disease activity. On the other hand, IH is an angiogenesis-related disease, in which polarized M2 and its cytokines substantially appear. Persistent activation of single polarized macrophages with their inflammatory mediators may closely implicate in the occurrence and development of those dermatoses; therefore, we propose that regulation of macrophage polarization direction would be a novel approach to treating these disorders.

### 5.1. M1 Polarization in Behcet's Disease (BD)

BD, a chronic recurrent multisystemic vascular inflammatory disease, is clinically characterized by oral aphthosis, genital ulcers, skin lesions, uveitis, and organ involvements [90, 91]. Patients with BD often suffer from relapsing painfully inflammatory attacks in involved organs. The histopathological feature of BD manifests as vasculitis with various inflammatory cell infiltrations and macrophages is a major one group of these inflammatory cells. Furthermore, the Th1-associated proinflammatory cytokines (e.g., TNF-*α*, INF-*γ*, IL-1*β*, IL-6) obviously emerged from the serum of active BD patients [92], which formed BD microenvironment that was regarded as a main inducing factor of BD. Later, Alpsoy et al. (2003) found in their experiment *in vitro* that the serum of BD patients could induce M1 macrophages [13]; meanwhile, study on BD-like mice model showed that the M1 highly expressed in BD-like mice compared with normal mice, followed by the increase of M1/M2 ratio in BD-like mice [43]. Besides, some M1-secreted factors, such as TNF-*α*, IL-1b, IL-6, IL-8, and IL-12, are closely associated with the disease activity [93, 94]. Up to now, it is thought that immune abnormality and oxidative damage are major players in BD pathogenesis. Relevant studies have demonstrated that T cell dysregulation, especially Th1 and Th17 expansions whereas Treg diminution, is partly responsible for BD [55, 95, 96]. Our previous studies, in the same way, confirmed that the OS-related parameters were significantly abnormal in BD patients, thereby considering that OS was one of the vital factors in BD pathogenesis. Furthermore, excessive ROS/RNS could promote the dysfunction of vascular endothelial cells in BD [62, 97]. These studies all indicate that there is a dominant M1 polarization in BD, which may further induce Th1/Th17 cell upregulation and cause OS damage. On the whole, M1 macrophages produce high-level proinflammatory cytokines and trigger the inflammatory events in BD [98, 99]; inflammatory factors from BD, in turn, recruit more M1 macrophages and aggravate inflammatory responses [13]. The current treatments for BD still display unsatisfactory efficacy; hence, macrophage polarization as a new therapeutic target would have important breakthroughs in its relief.

### 5.2. M1 Polarization in Psoriasis

Psoriasis is a chronic immune-mediated inflammatory disorder, histologically featuring as abnormal proliferation/differentiation of keratinocytes, excessive angiogenesis, and inflammatory cell infiltration in the dermis [100]. It has a great impact on the physical and psychological health of patients [101–103], but few therapeutic strategies are enough to satisfy. Thus, it is quite crucial to find the potential therapeutic targets for psoriasis. Emerging evidence supports that the recruitment and activation of macrophages in psoriatic skin lesions/blood vessels is a key pathogenic factor in the uncontrolled cutaneous/vascular inflammation [7, 104, 105]. A study from Lin et al. (2018) proved that a greatly high ratio of M1/M2 macrophages emerged from patients with severe psoriasis, and M1 in peripheral blood is absolutely superior to M2 [44]. Meanwhile, M1-related inflammatory cytokines obviously increase in psoriatic lesions, especially TNF-*α*. As a factor primarily derived from M1, TNF-*α* is considered as the master proinflammatory cytokine and is deemed to be a key candidate gene for the pathogenesis of psoriasis [106]. Apart from TNF-*α*, other M1-related proinflammatory cytokines (e.g., IL-6 and IL-1*β*) or chemokines (e.g., CXCL8) positively appear in psoriatic serum and facilitate inflammatory cell recruitment [107]. Although the exact mechanisms underlying initiation of psoriasis remain unclear, immune dysregulation and OS are mostly responsible for it. Th1/Th17 cell activation and Treg cell depletion could be triggered by M1, which in turn promote more M1 polarization and relatively suppress M2 activation via secreting IFN-*γ*, IL-17, and IL-23 [65]. The activation of IL-23/IL-17 axis due to dysregulation of Th1/Th17 cells is integral to the development of psoriasis, further to create a self-amplifying, feed-forward inflammatory response in keratinocytes [108]. On the one hand, the importance of ROS-induced OS in psoriasis was discussed in our previous document, namely, endogenous and exogenous factor-induced excessive ROS initiate OS that in turn promotes more ROS generation, further leading to immunological abnormality and the development of psoriasis [109]. Apart from that, ROS could induce the release of inflammatory factors that stimulate keratinocyte proliferation and angiogenesis, and directly damage the vascular endothelium as well as aggravate vascular inflammation. Above all indicate that M1 polarization is a key motivation to psoriasis; therefore, M1 polarization may be a potential therapeutic target for the treatment of psoriasis.

### 5.3. M1 Polarization in Systemic Lupus Erythematosus (SLE)

SLE, a multisystem autoimmune disease based on B cell immunity, involves the skin, connective tissue, and blood vessels [110]. It exhibits an unknown etiology with life-threatening manifestations. As a key part of the innate immune system, macrophages are probably responsible for SLE. Korman et al. (2014) favored that monocyte-to-macrophage differentiation could encourage the occurrence and development of SLE, possibly through polarizing macrophages towards M1 [111]. It has been long supposed that the imbalance between M1 and M2 is one of the possible causes of severe inflammation in SLE [18, 110, 112]. Evidence showed that several proinflammatory cytokines, e.g., TNF-*α*, GM-CSF, and IFN-*γ*, significantly elevated in the circulation of lupus patients, which formed a microenvironment to facilitate macrophage polarization towards M1 [113]. More importantly, many of the markers on M1 macrophages were found to remarkably ascend in SLE serum, which were implicated in the pathogenesis of SLE [114, 115]; for instance, BAFF, a member of TNF superfamily originating from M1 and an enhancement factor of M1 polarization [116, 117], primarily participates in stimulating B cell activation [118]; costimulatory molecules CD40 and CD86 also improve the ability of M1 to activate B cells and facilitate autoantibody production [119, 120]. Inflammatory cytokines, containing TNF-*α*, IL-1, IL-6, CXCL10, etc., obviously increase in SLE and positively stimulate M1 polarization [120, 121]. On the contrary, the failure in M2 polarization may be another crucial underlying mechanism in SLE. Studies show that macrophages in SLE patients exhibit low expression of CD163 (the marker molecule of M2), which indicates a defect of M2 polarization. It was confirmed in an experiment that adoptive transplantation of M2 not M1 macrophages significantly ameliorated the disease activity of SLE [112]. Although SLE is typically characterized by B cell hyperactivity, it may be aggravated in the presence of T cell dysregulation [122]. T cell dysregulation in SLE displays not only an abnormal activation of toxic cellular immunity due to Th1-Th17 cells elevation but also a failure of immune regulation from a decrease in Treg cells [123, 124]. Overactivated Th1/Th17 cells with their cytokines actively trigger a serial of pathological processes in SLE, including induction of vascular inflammation, recruitment of leukocytes, activation of B cells, and production of autoantibodies [125–127]; instead, the reduction of Tregs indicates the poor prognosis of SLE. In addition, ROS-induced OS damage is considered as another player in the pathogenesis of SLE and antioxidants targeting OS seem to be effective in SLE [128]. OS not only damages biomolecules to produce various autoantibodies in SLE [129, 130] but also contributes to vascular endothelial cell injury and triggers lupus vasculitis [63]. Anyhow, M1 overactivation with M2 absence would aggravate the imbalance between Th1/Th17 and Th2/Treg cells, and accelerate OS damage to cells/tissues, further accelerating the progression of SLE. Thus, regulation of M1/M2 polarization and enhancement of M2 anti-inflammatory molecules may slow the disorder progression in lupus models as well as in SLE patients [131, 132].

### 5.4. M2 Polarization in Infantile Hemangioma (IH)

As one of the most common benign tumors in infancy, IH is characterized by abnormal vascular endothelial cell proliferation and excessive angiogenesis; its pathogenesis involves genetic, environmental, and other factors [133]. This angiogenic disease is supposed to result from aberrant proliferation and differentiation of pluripotent progenitor cells. Generally, IH consists of three phases: proliferative phase, involuting phase, and involuted phase. The proliferating phase features as immature vascular mass [134, 135]. At present, it is believed that polarized M2 plays an important role in the proliferation period of hemangioma, which actively promotes the growth of vascular endothelial cell proliferation by secreting VEGF, FGF-2, and other proangiogenic factors [14, 15]. Hemangioma stem cells (HemSCs) are a class of stem cells with multiple differentiation potential in IH. Zhang et al. (2015) constructed a hemangioma mouse model *in vitro* through injecting HemSCs into the subcutaneous of mice, by which the differentiation potential of HemSCs was observed in a polarized M2 macrophage environment [136]. This finding indicates that M2 rather than M1 benefits the differentiation of HemSCs. Combined with previous research, a large number of CD163-positive cells, representing M2-polarized macrophages, were found surrounding the endothelial cells of proliferative IH and stimulating IH proliferation as well as VEGF/FGF signal transduction [14]. Accordingly, targeted regulation of M2 polarization during the proliferative phase of IH may be promising for management of IH.

## 6. Treatment Hotline

Considering the crucial role of dominant M1 or M2 in these dermatoses, various strategies are being explored to regulate the direction of their differentiation or block the transduction of downstream molecules, though most of them are still being in the pilot phase of cell or animal models. Here, we review the latest researches in the treatment of these vascular skin diseases by targeting macrophages, and more vehicles should be developed for modulating macrophage polarization in the future.

### 6.1. Progress in Therapies on Macrophage Polarization-Mediated Skin Diseases

Currently, many natural plant extracts or Chinese herbal ingredients have been widely studied on controlling macrophage polarization-mediated dermatosis owing to their multiple powerful activities, like anti-inflammation, immunoregulation, antioxidation, antiangiogenesis, and antitumor. It was reported that curcumin, a natural polyphenolic pigment derived from turmeric, could inhibit M1 polarization and decrease the level of IL-1*β*, IL-6, and TNF-*α* produced from M1 in BD patients [137]; what was more, curcumin induced anti-inflammatory M2 macrophage polarization and further mitigated the production of inflammatory cytokines [138]. Another study about curcumin treatment in a lupus mice model showed that curcumin markedly ameliorated lupus progression in mice, accompanied with a decrease of M1-derived BAFF production in the serum, spleen, and kidney as well as a reduction of activated B cells [139]. Similarly, a natural active compound from Astragalus membranaceus—cycloastragenol (CAG)—greatly ameliorated imiquimod- (IMQ-) induced psoriasiform dermatitis in mice by targeting regulation of proinflammatory macrophages (M1). CAG, on the one hand, obviously reduced the infiltration of M1 macrophages into psoriatic dermis; on the other hand, it in a dose-dependent mean lowered the level of proinflammatory cytokines (including IL-1*β*, TNF-*α*, and IL-6) in murine psoriatic skin and serum [140]. In addition, naringenin, a flavonoid compound, has been shown in a psoriasis-like mouse model to mitigate skin inflammation through accelerating the reprogramming of macrophages from M1 to M2 [141]. Although inflammatory cytokine antagonists have been gradually promoted in some autoimmune diseases recently, the application of exogenous anti-inflammatory factors is still limited to cell or animal models. For example, adalimumab as a TNF-*α* inhibitor could decrease the ratio of M1 to M2 macrophage in patients with psoriasis [44], while the cytokine IL-35 contributed to inhibiting the production of IL-6 and CXCL8 and alleviating the severe symptoms of IMQ-induced psoriasis-like mice via reducing the ratio of M1/M2 macrophages as well as the infiltration of M1 in local organs [142]. In the same way, Wu et al. (2017) suggested that local administration of TNF-*α* and IL-1*β*, produced from M1, would be a potential excellent option to IH through inducing endothelial-to-mesenchymal transition and accelerating hemangioma regression [89]. Moreover, some agents targeting the signaling pathways or receptors in macrophage polarization are undergoing an exploration in preliminary research. PAM3CSK4 (PAM3), a TLR2/1 agonist unlike other TLR agonists, could uniquely induce the conversion of lupus-patient monocytes to M2 *in vitro*; it was also shown that normal murine monocytes preferentially differentiated into M2 rather than M1 after weekly PAM3 treatment in the lupus-prone NZB/W mice (a mouse model of SLE whose symptoms and gender bias are similar to human) [132]. Thus, it raises the possibility of PAM3 being used to normalize the M1 : M2 ratio in SLE. Apart from above strategies, biomaterial application stands out. Recently, a promising study *in vitro* and in human psoriasis plaques has demonstrated that metallic polyphenol-enriched nanoparticles could not only reduce M1 but also suppress the production of proinflammatory cytokines, especially M1-derived TNF-*α* and IL-12; this new biological material, meanwhile, was found to powerfully repress NF-*κ*B (an important signal pathway of M1 polarization) activation in macrophages *in vitro* [143].

### 6.2. Future Orientation towards Macrophage Polarization-Mediated Dermatosis

**Figure 3:**
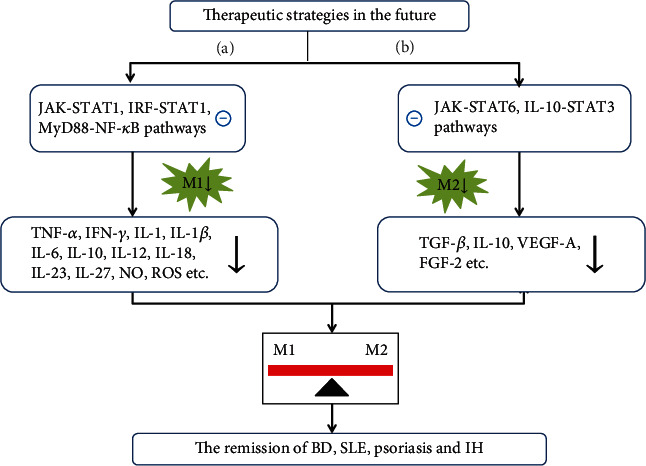
Future directions of treatment for macrophage polarization-mediated dermatosis. (a) Future treatment directions in M1 polarization-mediated skin diseases (BD, SLE, and psoriasis). (b) Future treatment directions in M2 polarization-mediated skin disease (IH). **㊀** indicates “inhibition” and ↓ indicates “downregulation”. Abbreviations: JAK: Janus kinase; STAT1/3/6: signal transducer and activator of transcription 1/3/6; IRF: interferon regulatory factor; IL-10: interleukin 10; MyD88: myeloid differentiation factor 88; NF-*κ*B: nuclear factor kappa B; M1: classically activated macrophage; M2: alternatively activated macrophage; TNF-*α*: tumor necrosis factor alpha; IFN-*γ*: interferon gamma; IL-1/1*β*/6/10/12/18/23/27: interleukin 1/1*β*/6/10/12/18/23/27; ROS: reactive oxygen species; TGF-*β*: transforming growth factor-*β*; NO: nitric oxide; VEGF-A: vascular endothelial growth factor A; FGF-2: fibroblast growth factor 2; SLE: systemic lupus erythematosus; BD: Behcet's disease; IH: infantile hemangioma.

According to the different phenotype macrophages and their respective roles in the dermatosis, some critical targets are proposed and summarized for the treatment of skin diseases via regulating macrophage polarization (shown in Figure 3). In the M1-dominant skin disorders (e.g., BD, SLE, and psoriasis), if blocking the upstream signals of M1 polarization (like JAK-STAT1, IRF-STAT1, and MyD88-NF-*κ*B), the level of M1 would decrease. Generally, polarized M1 can produce a lot of inflammatory mediators in serum (including TNF-*α*, IFN-*γ*, IL-1, IL-1*β*, IL-6, IL-10, IL-12, IL-18, IL-23, IL-27, NO, ROS). These inflammatory mediators from M1 are closely implicated in the occurrence and development of above dermatoses, so the exogenous intervention such as an addition of factor receptor antagonists may be a promising alternative. But in M2-dominant skin disorders, IH as the representative, the signal pathways involved in M2 polarization and the pathogenic molecules released from M2 are critical therapeutic targets for these diseases. Additional antagonists to hemangioma tissue contribute to the reversal of polarization state of M2 in IH via inhibition of M2 polarization-related signaling pathways (e.g., JAK/STAT6 and IL-10/STAT3) and downregulation of M2-related angiogenic factors (e.g., TNF-*β*, IL-10,VEGF-A, and FGF-2).

## 7. Conclusion

In conclusion, the effect of macrophage in different vascular dermatosis is variable owing to their M1/M2 phenotypes. Here, we present the evidence of macrophage polarization in M1/M2-mediated vascular skin diseases and discuss the possible pathological role of individual polarization types in these dermatoses. Typically in BD, psoriasis, and SLE, there is an enhancement in the ratio of M1/M2 and M1 plays a leading role in the continuous development and vicious cycle of inflammatory reactions, whereas in IH, the ratio of M1/M2 drops and the effect of M2 on angiogenesis are amplified. Targeting macrophage polarization to shift macrophages to M1 or M2 would be a novel strategy for control of vascular dermatosis; however, the mechanism of macrophage polarization in these diseases is not yet fully clarified and further studies in this field will be required. Besides, more therapeutic vehicles need to be developed and applied in clinical practice in the future.
